# SGLT2 Inhibition by Enavogliflozin Significantly Reduces Aβ Pathology and Restores Cognitive Function via Upregulation of Microglial AMPK Signaling in 5XFAD Mouse Model of Alzheimer's Disease

**DOI:** 10.1111/acel.70101

**Published:** 2025-05-10

**Authors:** Jihui Han, Jaehoon Song, Eun Sun Jung, Ji Won Choi, Hye Young Ji, Inhee Mook‐Jung

**Affiliations:** ^1^ Department of Biochemistry and Biomedical Sciences, College of Medicine Seoul National University Seoul Republic of Korea; ^2^ Convergence Dementia Research Center, College of Medicine Seoul National University Seoul Republic of Korea; ^3^ Life Science Institute Daewoong Pharmaceutical Yongin Republic of Korea

**Keywords:** Alzheimer's disease, amyloid‐beta, anti‐diabetic drugs, cognitive function, neuroinflammation, SGLT2 inhibition

## Abstract

Alzheimer's disease (AD) is a progressive neurodegenerative disorder characterized by cognitive decline. Metabolic dysfunctions, particularly type 2 diabetes mellitus (T2DM), have been implicated in AD pathogenesis, highlighting the potential for novel therapeutic approaches targeting shared underlying mechanisms. Here, we investigate sodium‐glucose cotransporter 2 (SGLT2) inhibition as a therapeutic strategy for AD using Enavogliflozin, a potent SGLT2 inhibitor, in the 5XFAD mouse model. Five‐month‐old 5XFAD mice were treated with Enavogliflozin (0.1 or 1 mg/kg) or vehicle for 8 weeks. The higher dose significantly improved cognitive performance in Y‐maze and Morris Water Maze tests, which correlated with enhanced synaptic plasticity and increased acetylcholine levels. Moreover, Enavogliflozin treatment reduced Aβ pathology and plaque burden, particularly affecting larger plaques. Mechanistically, SGLT2 inhibition attenuated neuroinflammation by suppressing NF‐κB signaling and proinflammatory cytokine production while promoting microglial recruitment to plaques. In vitro and ex vivo analyses further revealed that Enavogliflozin enhances microglial phagocytic capacity via AMPK‐mediated mitochondrial biogenesis and function. These findings highlight the multifaceted neuroprotective effects of SGLT2 inhibition in AD, demonstrating its potential to mitigate pathology and improve cognitive function. By uncovering its impact on neuroinflammation and microglial function, this study establishes SGLT2 inhibition as a promising therapeutic avenue for AD and other neurodegenerative disorders.

## Graphical abstract

SGLT2 inhibition with Enavogliflozin significantly improves cognitive function and reduces Aβ pathology in the 5XFAD mouse model of Alzheimer's disease. Through AMPK activation, Enavogliflozin enhances microglial mitochondrial function and Aβ phagocytosis while reducing neuroinflammation. These findings establish SGLT2 inhibitors as promising therapeutic candidates for Alzheimer's disease by modulating microglial metabolism to promote Aβ clearance and alleviate neurodegeneration.

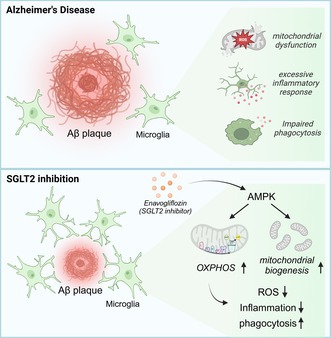

## 1 Introduction

Alzheimer's disease (AD) is a neurodegenerative disease characterized by gradual cognitive decline, memory loss, and behavioral changes. The pathological hallmarks of AD include the extracellular accumulation of amyloid‐beta (Aβ) plaques, intracellular neurofibrillary tangles composed of hyperphosphorylated tau protein, and chronic neuroinflammation (Selkoe and Hardy 2016). These pathological features lead to synaptic dysfunction, neuronal loss, and ultimately, cognitive impairment. Despite extensive research efforts, effective disease‐modifying treatments for AD remain elusive, underscoring the urgent need for novel therapeutic approaches that can address the complex, multifaceted nature of this disorder.

Numerous studies have suggested a strong link between type 2 diabetes mellitus (T2DM) and AD. Epidemiological studies have consistently demonstrated that individuals with T2DM have a higher risk of developing AD (Biessels and Despa 2018). This association reflects shared metabolic dysfunctions observed in both conditions, including insulin resistance, impaired glucose metabolism, and oxidative stress, all of which contribute to neuronal dysfunction and gradual cognitive decline (Craft 2009; de la Monte and Wands 2008; Park et al. 2023). Consequently, several anti‐diabetic agents are currently under investigation for their potential cognitive benefits in AD patients. For instance, glucagon‐like peptide‐1 (GLP‐1) receptor agonists, which enhance insulin secretion and reduce glucagon release, have shown promise in preclinical AD models and are now in clinical trials (Atri et al. 2022; Edison et al. 2021). Similarly, metformin, a widely used diabetes medication that regulates metabolic stress and bioenergetics, is being investigated for its potential neuroprotective effects in AD (Tang et al. 2024). These studies reflect a growing interest in targeting metabolic dysfunction as a therapeutic strategy for neurodegenerative disorders like AD.

Sodium‐glucose cotransporter 2 (SGLT2) inhibitors represent another class of anti‐diabetic drugs that have gained interest for their potential in AD treatment. The primary mechanism of action of SGLT2 inhibitors involves inhibiting glucose reabsorption in the proximal renal tubules, leading to increased urinary glucose excretion. This mechanism not only helps lower blood glucose levels independent of insulin but also promotes weight loss, addressing two key metabolic factors often associated with cognitive decline (Katsuumi et al. 2024). Preclinical studies have revealed potential neuroprotective properties of SGLT2 inhibitors beyond their metabolic effects. These include improvements in cognitive functions in diabetic mouse models, reduction of oxidative stress and neuroinflammation, and enhancement of neuronal plasticity and mitochondrial function in the brain (Pawlos et al. 2021; Rizzo et al. 2022). Furthermore, SGLT2 inhibitors have been shown to reduce hyperphosphorylated tau and Aβ accumulation while enhancing brain insulin signaling in a type 2 diabetes–AD mouse model (Sim et al. 2023). Such pleiotropic effects suggest that SGLT2 inhibitors may offer neuroprotective benefits beyond glycemic control, relevant to AD pathology.

Among the promising SGLT2 inhibitors, Enavogliflozin has emerged as a novel candidate for the management of T2DM (Kwak et al. 2023), with potential applications beyond diabetes management. While its efficacy in lowering blood glucose levels is well established, its potential effects under neurodegenerative conditions remain largely unexplored. However, preliminary studies with other SGLT2 inhibitors have demonstrated neuroprotective effects in various models of neurological disorders (Pawlos et al. 2021). These include reduced oxidative stress and neuroinflammation, improved blood–brain barrier (BBB) integrity, and enhanced synaptic connections. The observed benefits of other SGLT2 inhibitors in various neurodegenerative models suggest Enavogliflozin's potential neuroprotective properties in the context of AD pathology (Hierro‐Bujalance et al. 2020).

In this study, we investigated the therapeutic potential of SGLT2 inhibition against AD using the 5XFAD mouse model, which rapidly develops Aβ pathology and cognitive decline. We assessed cognitive outcomes following SGLT2 inhibition and explored the potential mechanisms underlying its observed neuroprotective effects, including changes in cholinergic signaling and synaptic plasticity. We also examined the effects of SGLT2 inhibition on Aβ deposition, plaque characteristics, and neuroinflammation, with a particular focus on microglial activity and its bioenergetics. Our findings demonstrate that Enavogliflozin significantly reduces Aβ deposition and neuroinflammation by enhancing microglial phagocytic activity via AMPK‐mediated mitochondrial modulation, leading to improved cognitive function in 5XFAD mice. By elucidating the mechanisms through which SGLT2 inhibition affects AD pathology and cognitive function, this study aims to provide valuable insights into the potential repurposing of SGLT2 inhibitors for AD treatment.

## 2 Materials and Methods

### 2.1 Animal

All animal experiments were approved by the Animal Care and Use Guidelines of Seoul National University, Korea (approval number: SNU‐230317‐1). The 5XFAD mouse model expresses five human mutations associated with AD under the regulation of the Thy1 promoter. These include three mutations in the human APP gene: Swedish (K670N/M671L), Florida (I716V), and London (V717I), as well as two mutations in the human PSEN1 gene (M146L and L286V). For drug administration, 5‐month‐old 5XFAD mice were orally administered vehicle or Enavogliflozin (Daewoong Pharm.) at 0.1 or 1 mg/kg daily for 8 weeks. Enavogliflozin was dissolved in the vehicle solution containing 0.5% carboxymethyl cellulose sodium salt (Sigma) and 1% Tween80 (Sigma). Body weights were measured every week, and drug dosages were adjusted to maintain a consistent mg/kg dose for each treatment group. After 8 weeks of administration, mice were sacrificed, and brain samples were collected for further analysis.

### 2.2 Oral Glucose Tolerance Tests

One day before the assay, the mice were fasted for 16 h. Mice were orally administered Enavogliflozin, followed by an intraperitoneal injection of glucose (2 g/kg). Blood samples were collected from the tail vein at 0, 30, 60, 120, and 180 min after glucose administration. Blood glucose levels were measured using a glucose analyzer (Roche). The area under the curve (AUC) for blood glucose concentration over the 180‐min sampling period was calculated using GraphPad Prism 8.0 software.

### 2.3 Behavioral Tests

#### 2.3.1 Y‐Maze Spontaneous Alternation Test

Spatial learning and short‐term memory were assessed using the Y‐maze test. Prior to testing, mice were habituated to the experimental room conditions for 1 h in darkness to alleviate anxiety. The Y‐maze consisted of three identical arms, each 35.5 cm long and 5 cm wide, arranged in a Y‐shape. Each mouse was allowed to explore the maze freely for 8 min. After each trial, the maze was thoroughly cleaned with 70% ethanol to eliminate olfactory cues that might influence subsequent trials. The sequence of arm entries was video‐recorded for subsequent analysis. An alternation was defined as consecutive entries into all three arms. The percentage of alternation was calculated as follows: [(number of alternations)/(total arm entries − 2)] × 100. To ensure the reliability of the results, behavioral analysis was conducted in a blinded manner.

#### 2.3.2 Morris Water Maze

A circular water maze with a diameter of 120 cm was filled with tap water at 23°C and made opaque with non‐toxic white paint. A hidden platform was submerged 1 cm below the water surface, positioned 30 cm from the tank wall in one quadrant. During the 5‐day training period, mice underwent four daily trials, starting from different cardinal positions: north, south, east, and west. Each trial lasted up to 60 s, and mice were guided to the platform if they failed to find it. Upon reaching the platform, mice remained there for 15 s, followed by 1‐min breaks between trials. On the test day, the platform was removed. Mice were placed in the center of the tank and allowed to swim for 60 s. All sessions were video‐recorded and analyzed using EthoVisionXT software (Noldus Information Technology). Parameters measured included swimming velocity, the number of platforms crossed, latency to first reach the platform, and time spent in the target quadrant during the probe trial. Between trials and after completion, mice were dried with paper towels and returned to their home cages. To ensure unbiased results, experimenters were blinded to the treatment conditions.

### 2.4 Western Blot

Brain tissue samples were homogenized in RIPA buffer supplemented with protease inhibitor cocktail, phosphatase inhibitor cocktails I and II, and PMSF (Sigma). The homogenates were sonicated and centrifuged at 13,000 rpm at 4°C for 15 min. Protein concentration in the collected supernatant was determined using a BCA protein assay. Samples were denatured at 95°C for 3 min and separated by SDS‐PAGE using NuPAGE 4%–12% Bis‐Tris gels (Invitrogen). Proteins were then transferred to PVDF membranes for immunoblotting. Membranes were blocked with 5% skim milk in TBST for 1 h at room temperature, followed by overnight incubation with primary antibodies at 4°C: SGLT2 (Abcam, #ab85626), BDNF (Abcam, #ab226843), APP (Biolegend, 6E10, #803001), phospho‐NFκB (Cell Signaling Technology, #3033), NFκB (Cell Signaling Technology, #8242), phospho‐AMPK (Cell Signaling Technology, #2535), AMPK (Cell Signaling Technology, #2532), VDAC1 (Invitrogen, #PA1‐954A), Tom20 (Santa Cruz, #11415), and β‐actin (Cell Signaling Technology, #3700). On the next day, membranes were washed with TBST five times and incubated with HRP‐conjugated secondary antibodies (Invitrogen) for 1 h. Protein bands were visualized using ECL reagents (Abfrontier) and captured with an Amersham Imager 600 (GE Healthcare Life Science). All protein levels were normalized to β‐actin, and densitometric analysis was performed using Multigauge software (Fujifilm Corporation).

### 2.5 Immunohistochemistry

Following 8 weeks of administration, mice were perfused with ice‐cold phosphate‐buffered saline (PBS). Brain samples were then extracted and fixed in 4% paraformaldehyde overnight. Subsequently, samples were immersed in 30% sucrose solution for 3 days for cryoprotection. Using a cryostat (Leica CM3050s), the samples were sectioned at 25–30 μm thickness. Antigen retrieval was performed by treating sections with 70% formic acid for 20 min. Sections were then washed with PBS, permeabilized, blocked in a solution of PBS containing 0.3% Triton X‐100 and 5% serum, and incubated with the following primary antibodies at 4°C overnight: PSD95 (Cell Signaling Technology, #36233, 1:250), 4G8 (Biolegend, #800704, 1:1000), Lamp1 (Abcam, #ab25245, 1:500), Iba1 (SY5Y, #234308, 1:1000), and GFAP (Invitrogen, #130300, 1:1000). The following day, sections were incubated with appropriate secondary antibodies for 1 h at room temperature. For Congo red staining, sections were incubated with 50 μM Congo red solution for 1 h at room temperature along with secondary antibodies. Sections were stained with DAPI (Sigma, 1:5000) for 10 min. Confocal images were acquired using the LSM700 Laser Confocal Microscope (Zeiss) and BC43 Microscope (Oxford Andor Technology) and analyzed using ImageJ software.

### 2.6 Enzyme‐Linked Immunosorbent Assay

Brain tissue samples were homogenized to measure specific biomarkers. Human Aβ40 and Aβ42 concentrations from the tissue homogenates were measured using human Aβ‐specific enzyme‐linked immunosorbent assay (ELISA) kits, according to the manufacturer's protocols (IBL 27713, 27711). Concentrations of TNFα and IL‐1β were determined using assay kits from R&D Systems (MTA00B, MLB00C) while acetylcholine levels were measured using a kit from Novus Biologicals (NBP2‐66389). All assays were performed following the protocols provided by the respective manufacturers.

### 2.7 Cell Viability Assay

Cell viability was assessed using the CellTiter 96 AQueous One Solution Cell Proliferation Assay (MTS, Promega #G3580). All assays were performed following the protocols provided by the respective manufacturers. Briefly, 20 μL reagent was added to each well containing 100 μL media and incubated at 37°C for 2 h in 5% CO_2_. Absorbance was measured at 490 nm.

### 2.8 Mitotracker and MitoSOX Staining

Cells were washed once with pre‐warmed PBS and incubated with MitoSOX reagent (Invitrogen, M36008) or MitoTracker (Invitrogen, M7514) for 30 min under light‐protected conditions. Following a PBS wash, cells were fixed with 4% paraformaldehyde for 15 min at room temperature. After three PBS washes, nuclei were counterstained with DAPI (1:5000 in PBS) for 10 min. Images were acquired using a bc43 Microscope (Oxford Andor Technology) and analyzed with ImageJ.

### 2.9 Seahorse

Oxygen consumption rate (OCR) was measured using a Seahorse XF24 analyzer (Seahorse Bioscience) according to the manufacturer's instructions. Briefly, 4 × 10^4^ cells were seeded onto XF24 cell culture microplates 24 h prior to analysis. The sensor cartridge was hydrated with calibrant buffer and equilibrated overnight at 37°C in a CO_2‐_free environment, 1 day before the assay. On the day of the assay, the culture medium was replaced with an assay medium (XF base medium supplemented with 1 mM pyruvate, 4 mM glutamine, and 25 mM glucose, pH 7.4), and cells were equilibrated at 37°C in a non‐CO_2_ incubator for 45–60 min. Mitochondrial function was evaluated using the Seahorse XF Cell Mito Stress Test Kit (Agilent, 103015‐100) with sequential injections of oligomycin (2 μM), FCCP (1 μM), and rotenone/antimycin A (0.5 μM). OCR were monitored continuously to quantify basal respiration, ATP production, maximal respiratory capacity, and non‐mitochondrial respiration.

### 2.10 Microglia Isolation From Mouse Brain (MACS)

PBS‐perfused brain tissue from 5XFAD mice, excluding the olfactory bulb and cerebellum, were mechanically dissociated with scissors and enzymatically digested in a solution containing 0.4% DNase I, 5% FBS, and 10 μM HEPES at 37°C for 15 min. Tissue homogenates were further dissociated by sequential trituration through serially narrowed Pasteur pipettes (10 strokes each). The resulting suspension was filtered through a 70‐μm cell strainer and centrifuged at 300 × g for 5 min at 4°C. The pelleted cells were gently resuspended in 3.1 mL ice‐cold DPBS, combined with 900 μL debris removal solution (Miltenyi Biotec, no. 130‐109‐398), and overlaid with 4 mL ice‐cold DPBS. Following centrifugation at 3000 × g for 5 min, a three‐phase separation was achieved. The upper two phases containing myelin debris were discarded. Half of the remaining cells were used for an ex vivo bead uptake assay, and the other half was used for microglia isolation. Microglia were subsequently isolated via positive selection using CD11b microbeads (Miltenyi Biotec, no. 130‐049‐601) and magnetic‐activated cell sorting (MACS). The purified microglial fraction was stored at −20°C until use.

### 2.11 Phagocytosis Assay (Bead Uptake Assay)

Microglial phagocytic activity was evaluated using fluorescent microsphere bead uptake assay. Fluoresbrite Yellow Green Carboxylate Microspheres (3.0 μm, Polysciences) were pre‐opsonized through incubation with DMEM containing 50% FBS for 30 min in a 37°C shaker. Primary microglial cultures were exposed to opsonized microspheres (1.0 × 10^6^ particles) in pre‐warmed DMEM and incubated for 1 h at 37°C in a humidified 5% CO_2_ incubator. Following incubation, cells were either immunolabeled with anti‐Iba1 antibody (SY5Y, #234308, 1:1000) for confocal microscopy (BC43) or detached for flow cytometry analysis (BD LSRFortessa X‐20).

### 2.12 Flow Cytometry

Cell suspensions were centrifuged (300 g, 5 min, 4°C) and resuspended in 50 μL FACS buffer. CD11b‐APC antibody (eBioscience, 17‐0112‐81, 1:1000) diluted in 50 μL FACS buffer was added to the cell suspension (total volume 100 μL) to label microglia and incubated for 30 min at 4°C in darkness. Cells were washed twice with 1 mL FACS buffer and centrifuged (300 g, 5 min, 4°C) between washes. For viability assessment, cells were incubated with Calcein Violet 405 (Invitrogen, C34858, 1:100) for 20 min. Stained samples were maintained at 4°C in darkness until analysis by flow cytometry.

### 2.13 RNA Isolation and Real‐Time Quantitative RT‐PCR

RNA was isolated using a QIAGEN RNeasy kit according to the manufacturer's protocol. cDNA was synthesized using Maxime RT Premix by reverse transcription (OligodT primer; Intronbio, 25081). Quantitative reverse‐transcription PCR (qRT‐PCR) conducted via real‐time PCR on the QuantStudio 3 (Applied Biosystems) using KAPA SYBR FAST reagents (Kapa Biosystems, KK4605). The following primers were used: *SGLT2* F: 5′‐CATTGGTGTTGGCTTGTGGT‐3′, *SGLT2* R: 5′‐GCGAACAGAGAGGCTCCAAC‐3′, *NLRP3* F: 5′‐CCCTTGGAGACACAGGACTC‐3′, *NLRP3* R: 5′‐GAGGCTGCAGTTGTCTAATC‐3′, *IL‐1β* F: 5′‐TGGCAACTGTTCCTG‐3′, *IL‐1β* R: 5′‐GGAAGCAGCCCTTCATCTTT‐3′, *IL‐18* F: 5′‐ACTGTACAACCGCAGTAATACGG‐3′, *IL‐18* R: 5′‐AGTGAACATTACAGATTTATCCC‐3′, *18s* F: 5′‐GTAACCCGTTGAACCCCATT‐3′, *18s* R: 5′‐CCATCCAATCGGTAGTAGCG‐3′.

### 2.14 Statistical Analysis

Statistical analyses were conducted using GraphPad Prism 8.0. Data are presented as means with standard error of the mean (SEM) indicated by error bars. For comparisons between two groups, a two‐tailed unpaired *t*‐test was used. When comparing more than two groups, one‐way analysis of variance (ANOVA) was performed, followed by Dunnett's post hoc test for multiple comparisons. Grubbs' test was used to identify outliers, with alpha set at 0.05. Statistical significance was defined as *p* < 0.05.

## 3 Results

### 3.1 Enavogliflozin Improves Glucose Tolerance in 5XFAD Mice

**FIGURE 1:**
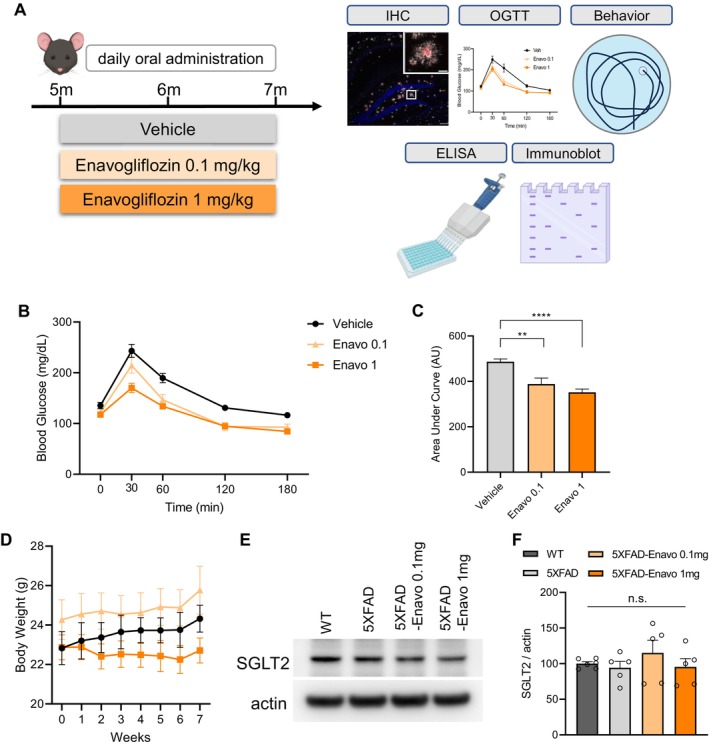
Enavogliflozin improves glucose tolerance in 5XFAD mice. (A) Schematic illustration of the experimental timeline. Starting at 5 months of age, 5XFAD mice were orally administered vehicle, 0.1 mg/kg, or 1 mg/kg Enavogliflozin daily for 2 months. (B) Oral glucose tolerance test (OGTT) performed after 16 h of fasting at the end of the 2‐month treatment period. Mice were administered a glucose solution (2 g/10 mL/kg) following Enavogliflozin treatment, and blood glucose levels were measured at indicated time points using a glucometer. (C) Area under the curve (AUC) calculated from the OGTT data in (B), *n* = 10–15 per group. **p < 0.01, ****p < 0.0001. (D) Body weights measured weekly throughout the 8‐week treatment period, *n* = 10 per group. (E) Representative Western blot images showing SGLT2 protein levels in wildtype (WT) and 5XFAD mice with or without Enavogliflozin treatment. (F) Densitometric analysis of SGLT2 protein levels normalized to β‐actin, *n* = 5 per group, n.s., not significant. Data are presented as mean ± SEM. Statistical analysis was performed using one‐way ANOVA followed by Tukey's post hoc test.

Neuronal loss and cognitive decline in 5XFAD mice typically begin at 5 to 6 months of age (Oakley et al. 2006). To investigate the neuroprotective potential of SGLT2 inhibition against AD pathology, we administered Enavogliflozin, a potent SGLT2 inhibitor, to 5XFAD mice beginning at 5 months of age. Mice were orally administered Enavogliflozin at two doses, 0.1 and 1 mg/kg, for 2 months, after which cognitive and pathological changes were evaluated (Figure 1A). To confirm the pharmacological efficacy of Enavogliflozin in the 5XFAD model throughout the intervention period, we performed an Oral Glucose Tolerance Test (OGTT) at the end of the treatment to assess glucose metabolism. Following a 16‐h fasting period, blood glucose concentrations were measured at baseline (0 min) and at 30, 60, 120, and 180 min post‐glucose administration (Figure 1B). Quantitative analysis of the AUC for glucose concentrations revealed a significant dose‐dependent reduction in the Enavogliflozin‐treated groups compared to vehicle controls (Figure 1C). These alterations in glucose dynamics demonstrate that Enavogliflozin effectively modulated glucose homeostasis in the 5XFAD model, confirming its intended pharmacological mechanism of action. Throughout the 8‐week administration period, we monitored body weight weekly to assess potential metabolic effects resulting from either SGLT2 inhibition or AD progression. Although weight loss has been reported in aged 5XFAD mice (Gendron et al. 2021), we did not observe significant weight reduction attributable to AD pathology progression in our vehicle‐treated 5XFAD under the current experimental settings. However, consistent with the established metabolic effects of SGLT2 inhibitors, we documented a significant attenuation of body weight gain in the 1 mg/kg Enavogliflozin group relative to vehicle controls throughout the intervention period (Figure 1D). Western blot analysis revealed that these physiological effects occurred without alterations in SGLT2 expression levels (Figure 1E,F), suggesting that Enavogliflozin exerted its metabolic effects without directly affecting its expression level (Figure 1E,F).

### 3.2 SGLT2 Inhibition Enhances Cognitive Function and Synaptic Plasticity

**FIGURE 2:**
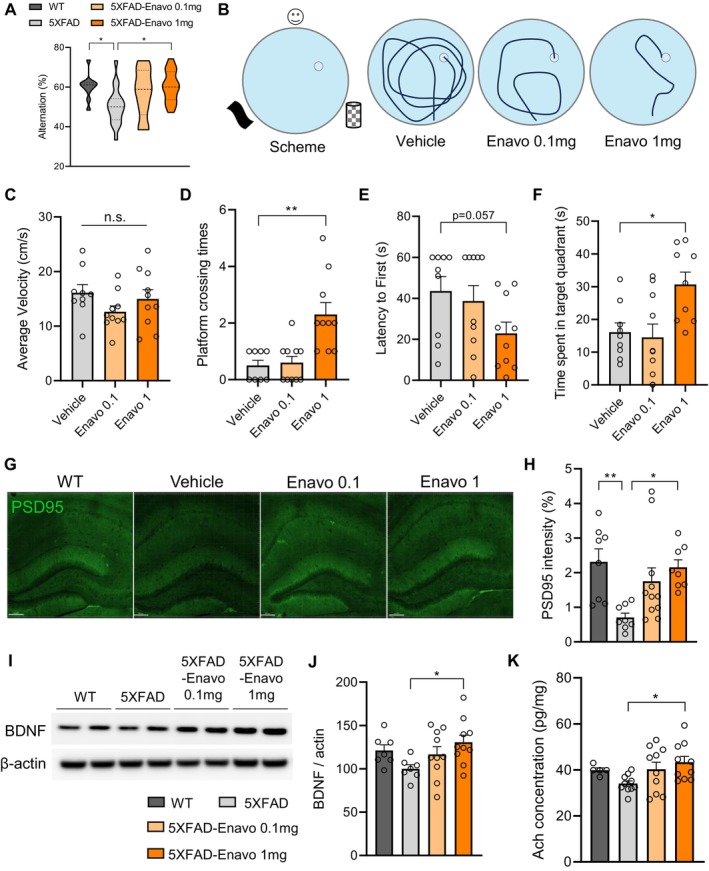
SGLT2 inhibition enhances cognitive function and synaptic plasticity. (A) Percentage of spontaneous alternation in the Y‐maze test, calculated as the number of alternations divided by the total arm entries. *p < 0.05. (B) Schematic representation of the Morris Water Maze apparatus with a platform positioned in one quadrant. Representative swim path traces from different treatment groups 24 h after the last training session. (C) Average swimming velocity during the probe test to assess motor function, n.s., not significant. (D) Number of platform crossings during the 60‐s probe test. **p < 0.01. (E) Latency to first platform crossing during the probe test. (F) Time spent in the target quadrant during the 60‐s probe test. *p < 0.05. (G) Representative confocal microscopy images of hippocampal sections immunostained for PSD95 (green) in 7‐month‐old 5XFAD mice following 2 months of treatment. Scale bar = 150 μm. (H) Quantification of PSD95 intensity *p < 0.05, **p < 0.01. (I) Representative Western blot images showing BDNF protein levels in 7‐month‐old 5XFAD mice after 2 months of treatment. (J) Densitometric analysis of BDNF protein levels normalized to β‐actin, *p < 0.05. (K) Acetylcholine (ACh) concentration in tissue homogenates measured by ELISA, *p < 0.05. All analysis was performed with 10–15 mice per group. Data are presented as mean ± SEM. Statistical analysis was performed using one‐way ANOVA followed by Tukey's post hoc test.

To evaluate the cognitive effects of SGLT2 inhibition, we conducted Y‐maze and Morris Water Maze (MWM) behavioral assessments. Y‐maze revealed that the working memory deficits characteristic of 5XFAD mice were significantly restored in the 1 mg/kg Enavogliflozin‐treated group (Figure 2A). We next conducted the Morris Water Maze test to assess spatial learning and memory. With the hidden platform positioned in the northeast quadrant, mice underwent five consecutive days of acquisition training. Representative swim path traces revealed a longer distance to find the platform in the vehicle group, whereas Enavogliflozin‐administered groups showed decreased distance and increased navigation ability to the platform (Figure 2B). While locomotor capacity remained consistent across all experimental groups, as evidenced by comparable swimming velocities (Figure 2C), the probe test revealed marked improvements in spatial memory in the 1 mg/kg Enavogliflozin group. This group demonstrated a significant increase in platform crossing times, decreased latency to the first platform crossing, and spent significantly more time in the target quadrant compared to the vehicle group (Figure 2D–F). Meanwhile, the 0.1 mg/kg dose was insufficient to induce a statistically significant change in learning and memory, indicating a dose‐dependent therapeutic threshold (Figure 2A–F). These results indicate that Enavogliflozin treatment at 1 mg/kg significantly enhanced spatial learning and memory in the 5XFAD mouse model, suggesting a cognitive benefit in AD.

To elucidate the molecular substrates underlying these cognitive enhancements, we investigated synaptic integrity markers and cholinergic signaling. Immunofluorescence analysis of postsynaptic density protein 95 (PSD95) demonstrated that nearly complete rescue of impaired synaptic health in 5XFAD by 1 mg/kg Enavogliflozin treatment (Figure 2G,H). Complementary Western blot analyses revealed significantly elevated expression of brain‐derived neurotrophic factor (BDNF) in the high‐dose treatment group (Figure 2I,J), a critical mediator of activity‐dependent synaptic plasticity and cognitive function. Furthermore, we examined cholinergic transmission, which reflects synaptic health and is highly relevant in AD. A deficit in cholinergic neurotransmission is prevalent in AD (Cummings et al. 2024), and acetylcholinesterase (AChE) inhibitors such as donepezil, rivastigmine, and galantamine are widely prescribed as cognitive enhancers to augment cholinergic signaling and improve cognitive performance in AD patients (Marucci et al. 2021). Interestingly, several SGLT2 inhibitors, including Canagliflozin and Empagliflozin, have been reported to exhibit AChE inhibitory properties (Shakil 2017). Consistent with these reports, we observed a significant increase in acetylcholine levels in the high‐dose Enavogliflozin group to levels comparable with wild‐type controls (Figure 2K). Collectively, the increased synaptic molecules and enhanced cholinergic neurotransmission provide a potential molecular mechanism for cognitive improvements by SGLT2 inhibition.

### 3.3 SGLT2 Inhibition Reduces Aβ Pathology in 5XFAD Mice

**FIGURE 3:**
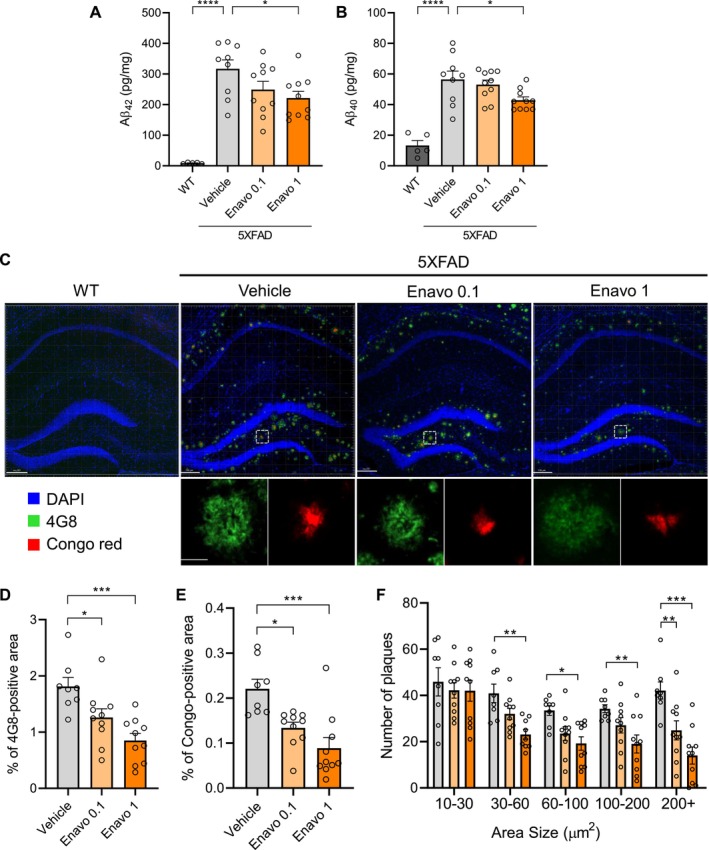
SGLT2 inhibition reduces Aβ pathology in 5XFAD mice. (A, B) Quantification of Aβ42 (A) and Aβ40 (B) concentrations in brain tissue homogenates measured by ELISA, *n* = 5–10 per group, *p < 0.05, ****p < 0.0001. (C) Representative confocal microscopy images of hippocampal sections immunostained with DAPI (blue) for nuclei, 4G8 (green) for total Aβ, and Congo red (red) for Aβ plaque cores. Upper panels show whole hippocampal images (scale bar = 150 μm), and lower panels show magnified images of the boxed areas (scale bar = 10 μm). (D) Quantification of 4G8‐positive area. *p < 0.05, ***p < 0.001. (E) Quantification of Congo red‐positive area. *p < 0.05, ***p < 0.001. (F) Number of plaques categorized by size ranges (10–30, 30–60, 60–100, 100–200, and > 200 μm^2^). *p < 0.05, **p < 0.01, ***p < 0.001. All analyses were performed with 9–10 mice per group. Data are presented as mean ± SEM. Statistical analysis was performed using one‐way ANOVA followed by Dunnett's post hoc test.

Progressive accumulation of Aβ is a key neuropathological feature of AD and a primary target for therapeutic intervention. To evaluate the potential of SGLT2 inhibition as a therapeutic strategy, we examined its effects on Aβ pathology. Quantification of Aβ isoforms using ELISA revealed a significant reduction in both Aβ42 and Aβ40 levels following Enavogliflozin treatment, with the most pronounced effect observed in the 1 mg/kg group (Figure 3A,B). To further assess Aβ deposition, we performed immunofluorescence staining of the hippocampus using the 4G8 antibody, which recognizes residues 17–24 of the Aβ sequence, and Congo red, which selectively binds to β‐sheet‐rich plaque cores. Co‐staining revealed that Congo red fluorescence was concentrated at the center of 4G8‐positive regions, indicative of dense‐core plaques (Figure 3C). Quantitative analysis demonstrated a significant, dose‐dependent reduction in both 4G8‐positive and Congo red‐positive areas following Enavogliflozin treatment (Figure 3D,E). Additionally, the total number and average size of plaques were significantly reduced in the 1 mg/kg treatment group (Figure S1A,B). Interestingly, the reduction in plaque number was more pronounced in larger plaques, whereas smaller plaques (< 30 μm^2^) remained unchanged across all groups (Figure 3F). Since Enavogliflozin treatment did not affect amyloid precursor protein (APP) expression (Figure S1C), we hypothesized that SGLT2 inhibition reduces Aβ plaques primarily by modulating Aβ aggregation and clearance rather than affecting APP levels. To investigate the impact of SGLT2 inhibition on neuronal health, we performed immunofluorescence staining for LAMP1, a marker of dystrophic neurites, and 4G8 (Huang and Lemke 2022). Notably, LAMP1 signals associated with Aβ plaques were significantly reduced in the 1 mg/kg Enavogliflozin‐treated group, suggesting a decrease in dystrophic neurites. This reduction in neurite dystrophy implies a potential alleviation of Aβ‐induced neuronal damage. Collectively, these findings demonstrate that SGLT2 inhibition via Enavogliflozin treatment effectively mitigates Aβ pathology in the 5XFAD mouse model, reducing both Aβ burden and plaque size while alleviating plaque‐associated neuritic damage.

### 3.4 SGLT2 Inhibition Attenuates Pro‐Inflammatory Phenotypes and Enhances Microglial Recruitment for Aβ Phagocytosis

**FIGURE 4:**
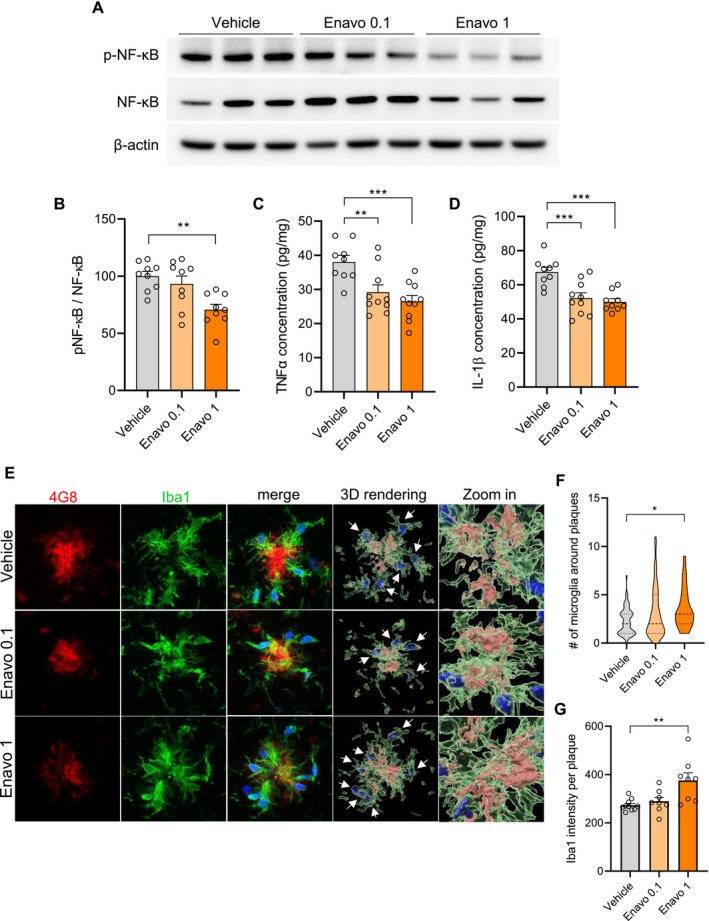
SGLT2 inhibition attenuates pro‐inflammatory phenotype and enhances microglial recruitment for Aβ phagocytosis. (A) Representative Western blot images showing phosphorylated NF‐κB (p‐NF‐κB) and total NF‐κB protein levels in brain tissue homogenates from 5XFAD mice after 2 months of treatment with vehicle or Enavogliflozin (0.1 or 1 mg/kg). (B) Densitometric analysis of p‐NF‐κB to total NF‐κB ratio, **p < 0.01. (C) TNFα concentration in brain tissue homogenates measured by ELISA, **p < 0.01, ***p < 0.001. (D) IL‐1β concentration in brain tissue homogenates measured by ELISA, ***p < 0.001. (E) Representative confocal microscopy images showing 4G8 (red), Iba1 (green), and DAPI (blue) staining. 3D rendering images demonstrate IMARIS rendering of Iba1‐positive microglia and 4G8‐positive Aβ deposits within the microglia. White arrows indicate Iba1‐positive microglia surrounding 4G8‐positive Aβ. Scale bar = 10 μm. (F) Quantification of the number of microglia around Aβ plaques. *p < 0.05. (G) Quantification of Iba1 intensity around Aβ deposits, **p < 0.01. All analyses were performed with 9–10 mice per group. Data are presented as mean ± SEM. Statistical analysis was performed using one‐way ANOVA followed by Tukey's post hoc test.

Neuroinflammation represents a critical aspect of AD pathogenesis. We therefore investigated the effects of Enavogliflozin on gliosis and related inflammatory markers. Immunofluorescence staining revealed a dose‐dependent reduction in Iba1‐positive microgliosis in Enavogliflozin‐treated groups (Figure S2A,B), with a parallel decrease in GFAP‐positive astrocytosis (Figure S2C,D). To characterize changes in pro‐inflammatory glial phenotypes, we examined key components of the NF‐κB signaling pathway, which is central to inflammatory responses in microglia. Western blot analysis demonstrated a significant reduction in NF‐κB phosphorylation, normalized to total NF‐κB levels, in the 1 mg/kg Enavogliflozin group (Figure 4A,B). Since NF‐κB activation induces expression of pro‐inflammatory cytokines, we measured levels of TNFα and IL‐1β using ELISA. Consistent with the observed reduction in NF‐κB activation, both TNFα and IL‐1β levels exhibited dose‐dependent decreases upon Enavogliflozin treatment (Figure 4C,D). Collectively, these reductions in gliosis, pro‐inflammatory signaling, and downstream cytokines provide robust evidence for the anti‐inflammatory effects of SGLT2 inhibition in the 5XFAD mouse model.

To elucidate the relationship between inflammation and Aβ pathology in our experimental paradigm, we conducted correlation analyses between Iba1‐ and GFAP‐positive areas with 4G8‐positive Aβ plaque areas (Figure S3A,B). Notably, only microglia, not astrocytes, demonstrated a strong correlation with Aβ plaque burden (*R* ^2^ = 0.7946, *p* < 0.0001). This observation prompted further investigation into the role of microglia in modulating Aβ pathology. Following phagocytosis of Aβ, microglia condense Aβ into dense core plaques characterized by highly compacted Aβ fibrils, resulting in reduced neurotoxicity (Huang et al. 2021). Consistent with this mechanism, we observed increased microglial recruitment around plaques in the 1 mg/kg Enavogliflozin‐treated group (Figure 4E–G). In conjunction with the significant reduction in large‐sized plaques (Figure 3F), these findings highlight microglia as the predominant modulators of Aβ pathology under SGLT2 inhibition, specifically affecting plaque compaction and enhancing Aβ phagocytosis, thereby reducing the overall inflammatory burden in the 5XFAD model.

### 3.5 Enavogliflozin Increases Mitochondrial Biogenesis and Function Through AMPK Signaling

**FIGURE 5:**
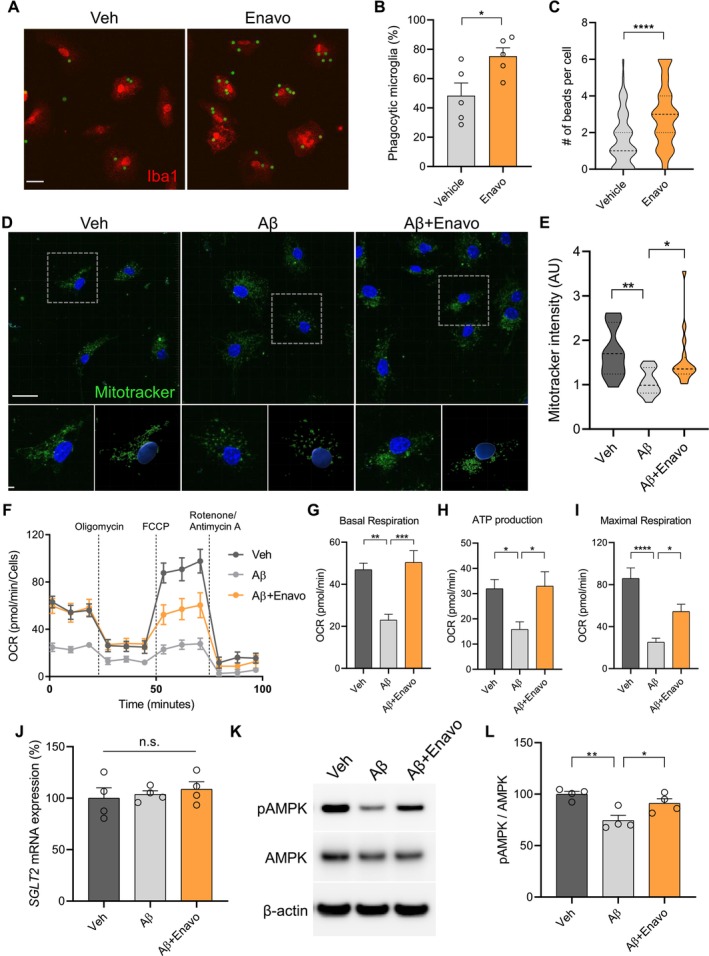
Enavogliflozin increases mitochondrial biogenesis and function through AMPK signaling. (A) Representative confocal microscopy images of primary microglia (Iba1, red) treated with vehicle or Enavogliflozin (0.1 μM) for 24 h, followed by bead (FITC) uptake for 1 h. Scale bar = 10 μm. (B) Quantification of phagocytic microglia, defined as Iba1‐positive cells containing more than four beads, expressed as percentage of total microglia, from 5 independent experiments. *p < 0.05. (C) Quantification of the number of phagocytosed beads per microglial cell, from 5 independent experiments. ****p < 0.0001. (D) Representative confocal microscopy images of primary microglia treated with vehicle, Aβ (4 μM), or Aβ with Enavogliflozin (0.1 μM) for 24 h and stained with Mitotracker (green). Scale bar = 10 μm. (E) Quantification of Mitotracker intensity, from 4 independent experiments. *p < 0.05, **p < 0.01. (F) Oxygen consumption rate (OCR) measured by Seahorse assay in primary microglia treated with vehicle, Aβ (4 μM), or Aβ with Enavogliflozin (0.1 μM) for 24 h. Oligomycin, an inhibitor of ATP synthase; carbonyl cyanide‐p‐trifluoromethoxyphenylhydrazone (FCCP), the reversible inhibitor of OXPHOS; rotenone/antimycin A (Rote/AA), the mitochondrial complex I and complex III inhibitor. Cells were treated sequentially with 2 μM oligomycin, 1 μM FCCP, and 0.5 μM Rote/AA, as indicated. (G–I) Quantification of basal respiration (G), ATP production (H), and maximal respiration (I) from the OCR measurements from 4 independent experiments. *p < 0.05, **p < 0.01, ***p < 0.001, ****p < 0.0001. (J) SGLT2 mRNA expression in primary microglia treated with vehicle, Aβ, or Aβ with Enavogliflozin measured by qRT‐PCR, from 4 independent experiments. n.s., not significant. (K) Representative Western blot images showing phosphorylated AMPK (pAMPK), total AMPK, and β‐actin. (L) Densitometric analysis of pAMPK to AMPK ratio from 4 independent experiments. *p < 0.05, **p < 0.01. Data are presented as mean ± SEM. Statistical analysis was performed using one‐way ANOVA followed by Tukey's post hoc test.

To elucidate the molecular mechanism underlying microglia‐mediated Aβ plaque modulation by Enavogliflozin, we investigated microglial phagocytosis, a process critical for Aβ clearance. Given that phagocytosis is an energy‐intensive process requiring robust mitochondrial function (Fairley et al. 2023), we hypothesized that Enavogliflozin enhances microglial phagocytic capacity through mitochondrial regulation. We first established the optimal concentration range for Enavogliflozin in primary microglia culture using MTS viability assays. The assay demonstrated that Enavogliflozin maintained cellular viability at concentrations up to 10 μM, with significant cytotoxicity observed from 30 μM (Figure S4). Thus, the subsequent experiments were conducted at 0.1 μM. Using this established non‐cytotoxic concentration, we proceeded to assess the effects of Enavogliflozin on microglial phagocytic function. Fluorescent bead uptake assays revealed significant increases in both the percentage of phagocytic microglia and the phagocytic capacity per cell following Enavogliflozin treatment (Figure 5A–C). Mitochondrial mass, visualized by Mitotracker immunofluorescence, was significantly elevated in Enavogliflozin‐treated microglia compared to Aβ‐exposed cells (Figure 5D,E). Moreover, seahorse metabolic flux analysis demonstrated that Enavogliflozin ameliorated Aβ‐induced mitochondrial dysfunction, as evidenced by restoration of basal respiration, maximal respiratory capacity, and ATP production (Figure 4F–I). Furthermore, mitochondrial oxidative stress, analyzed by MitoSOX fluorescence, was significantly increased following Aβ exposure but was markedly attenuated with Enavogliflozin co‐treatment (Figure S5).

Meanwhile, treatment with Enavogliflozin restored AMPK phosphorylation, which was downregulated by Aβ, without altering SGLT2 transcriptional levels (Figure 5K,L). Given AMPK's established role as a master regulator of mitochondrial biogenesis and function (Herzig and Shaw 2018), our findings suggest that SGLT2 inhibition enhances microglial phagocytic activity via AMPK‐dependent mitochondrial regulation. To validate the physiological relevance of this mechanism, we conducted ex vivo cultures of microglia isolated from 5XFAD mice (Figure S6A). Consistent with our primary culture findings, Enavogliflozin treatment increased AMPK phosphorylation without affecting SGLT2 expression (Figure S6B,C) and upregulated the mitochondrial proteins VDAC1 and Tom20 (Figure S6D–F). Furthermore, Enavogliflozin enhanced microglial phagocytic activity (Figure S6G) while simultaneously reducing inflammatory phenotypes, as assessed by decreased expression of NLRP3 inflammasome components and pro‐inflammatory cytokines IL‐1β and IL‐18 (Figure S6H).

## 4 Discussion

In this study, we demonstrated that Enavogliflozin, a potent SGLT2 inhibitor, effectively ameliorates multiple aspects of AD pathology in the 5XFAD mouse model. Daily oral administration of Enavogliflozin for 2 months not only confirmed its efficacy through improved glycemic control and attenuated body weight gain, but also revealed significant therapeutic effects on AD pathogenesis. Most notably, Enavogliflozin treatment led to dose‐dependent reductions in hippocampal Aβ plaque burden, decreased neuroinflammation, and improved cognitive function. These comprehensive benefits suggest potential disease‐modifying effects of SGLT2 inhibition that extend beyond glucose regulation.

The reduction in Aβ plaque burden observed with SGLT2 inhibition provides compelling evidence for its therapeutic potential. Our detailed analysis revealed that Enavogliflozin treatment induced a significant reduction in both Aβ40 and Aβ42 levels, decreased plaque size, and notably diminished the number of large plaques (> 60 μm^2^) while smaller plaques remained relatively unchanged. Furthermore, the reduction in LAMP1‐positive dystrophic neurites associated with Aβ plaques indicates that Enavogliflozin treatment mitigates plaque‐induced neuronal damage, a critical aspect of AD pathology. The significant correlation between reduced plaque burden and microglial activation indicates that Enavogliflozin may attenuate AD pathology primarily via modulation of microglial response. Numerous studies have highlighted the complex role of neuroinflammation in AD pathogenesis (Heneka et al. 2015; Kinney et al. 2018). Our observation of reduced NF‐κB signaling and decreased pro‐inflammatory cytokines (TNFα and IL‐1β) in Enavogliflozin‐treated groups provides insight into the drug's anti‐inflammatory mechanisms. These findings are consistent with previous studies demonstrating the anti‐inflammatory effects of SGLT2 inhibitors in various disease models (Ganbaatar et al. 2020; Heerspink et al. 2019).

Beyond attenuating pro‐inflammatory phenotypes, our results demonstrate that SGLT2 inhibition enhances microglial phagocytic capacity through AMPK‐dependent mitochondrial regulation. Using both in vitro and ex vivo approaches, we found that Enavogliflozin restored AMPK phosphorylation that was downregulated by Aβ exposure. This activation of AMPK signaling led to increased mitochondrial biogenesis and improved mitochondrial function, leading to enhanced microglial phagocytic activity, with increased uptake of fluorescent beads and greater microglial recruitment around Aβ plaques in Enavogliflozin‐treated mice. This aligns with the growing recognition of AMPK as a critical therapeutic target in neurodegenerative diseases. Interestingly, multiple studies have reported that SGLT2 inhibitors activate the AMPK signaling pathway (Hawley et al. 2016; Lee et al. 2021). AMPK functions as a central metabolic sensor that orchestrates cellular responses to stress, and its dysregulation has been implicated in AD pathogenesis (Herzig and Shaw 2018). The relationship between AMPK and mitochondrial function in microglia deserves particular attention, as mitochondria in immune cells serve not only as powerhouses for cellular metabolism but also as signaling platforms that coordinate inflammatory responses (Weinberg et al. 2015). Our findings demonstrating Enavogliflozin's ability to enhance mitochondrial mass, reduce oxidative stress, and improve respiratory capacity via AMPK activation provide mechanistic insight into how SGLT2 inhibition may restore microglial phagocytic capacity, thereby facilitating Aβ clearance and attenuating neuroinflammation.

While we observed a significant reduction in astrocyte activation, this did not correlate directly with Aβ levels, suggesting distinct mechanisms by which Enavogliflozin affects astrocytes and microglia in the context of AD pathology (Hasel et al. 2023). Previous studies have highlighted complex interactions between astrocytes, Aβ, and tau pathology. A study using primary cultures demonstrated that astrocytes exacerbate Aβ‐induced neuronal death and are essential for Aβ‐induced tau phosphorylation (Garwood et al. 2011). Clinical studies further support this, showing an Aβ association with increased plasma phosphorylated tau only in individuals with astrocyte reactivity (Bellaver et al. 2023). These findings suggest that the Enavogliflozin‐induced reduction in astrocyte activation observed in our study could have broader implications on tau pathology and neuronal survival, aspects not directly observable in our 5XFAD model. This highlights potential avenues for future research in models exhibiting both Aβ and tau pathology.

The cognitive improvements observed with SGLT2 inhibition, particularly in spatial learning and memory, were accompanied by significant molecular changes that provide insight into potential mechanisms of action. Our findings revealed increased synaptic integrity, as evidenced by enhanced PSD95 expression, alongside elevated acetylcholine levels. This enhancement of cholinergic signaling is particularly relevant given that cholinergic dysfunction is a hallmark of AD, and traditional FDA‐approved treatments primarily target this system through acetylcholinesterase inhibition. The demonstration that Enavogliflozin can modulate cholinergic signaling, similar to other SGLT2 inhibitors, suggests shared molecular targets with traditional FDA‐approved AD treatments (Khamies et al. 2024; Pang et al. 2023). In addition, we observed significant upregulation of BDNF expression in Enavogliflozin‐treated groups. BDNF plays crucial roles in neurogenesis (Zigova et al. 1998), memory persistence (Bekinschtein et al. 2008), and glial activation (Ding et al. 2020). The increased expression of BDNF observed in this study might mediate the multi‐dimensional beneficial effects of Enavogliflozin.

The high potency of Enavogliflozin compared to other SGLT2 inhibitors represents a significant advantage for potential therapeutic applications. Clinical trials have demonstrated that 0.3 mg/day of Enavogliflozin achieves anti‐diabetic effects comparable to 10 mg/day of Dapagliflozin (Sohn et al. 2024). This superior potency could translate to lower effective doses in humans, potentially reducing side effects and improving tolerability. Furthermore, our finding that 1 mg/kg/day of Enavogliflozin mitigated cognitive decline in 5XFAD mice is particularly promising when compared to studies using higher doses (10 mg/kg/day) of other SGLT2 inhibitors such as Empagliflozin or Canagliflozin in AD models (Hierro‐Bujalance et al. 2020; Khamies et al. 2024).

An important consideration in evaluating Enavogliflozin's efficacy for AD is its ability to cross the BBB. While previous studies in ICR mice showed minimal brain penetration of Enavogliflozin (0.04%), our study with 5XFAD mice revealed a significantly higher brain tissue distribution of 1.3% and an average concentration at 9.95 nM (data not shown). This 32.5‐fold increase in penetration likely reflects the altered BBB permeability associated with AD pathology in 5XFAD mice (Liu et al. 2020). The compromised BBB integrity commonly observed in AD may facilitate greater central nervous system exposure to peripherally administered drugs (Sweeney et al. 2018). This enhanced brain penetration could explain the robust central effects we observed and suggests that AD‐related BBB changes can facilitate drug delivery to affected brain regions.

The potential repurposing of SGLT2 inhibitors for AD treatment offers significant advantages in terms of time and cost compared with de novo drug development. Among anti‐diabetic drugs, SGLT2 inhibitors are considered relatively safe, with minimal side effects (McGill and Subramanian 2019). Recent population‐based studies have shown encouraging results, with a large cohort study demonstrating reduced risk of dementia in SGLT2 inhibitor users compared to DPP‐4 inhibitor users, particularly after 2 years of treatment (Shin et al. 2024). Supporting these findings, other studies have reported reduced consumption of AD medications among patients prescribed SGLT2 inhibitors compared to those on other anti‐diabetic agents (Zamora et al. 2024). Furthermore, longitudinal studies have demonstrated improved cognitive function during long‐term SGLT2 inhibitor use, supporting their potential therapeutic value in AD treatment (Low et al. 2022). These epidemiological findings, combined with our mechanistic insights, suggest that SGLT2 inhibitors, particularly potent ones like Enavogliflozin, represent a promising therapeutic strategy for AD, though randomized controlled trials are needed to confirm their efficacy.

Although our study presents compelling evidence for the protective effects of SGLT2 inhibition against AD, several limitations need to be addressed. First, the relative contributions of systemic effects versus direct brain effects remain unclear. While our BBB penetration data suggest direct central nervous system effects of SGLT2 inhibition, the degree to which peripheral metabolic improvements contribute to the observed benefits requires further investigation. Future studies employing cell‐type specific SGLT2 knockout models or brain‐specific drug delivery systems could help distinguish between central and peripheral mechanisms of action. Second, although we observed significant effects on Aβ pathology and neuroinflammation, our 5XFAD model does not exhibit significant tau pathology, another critical factor in AD pathogenesis. Previous studies with other SGLT2 inhibitors have shown reduced levels of hyperphosphorylated tau in different AD models, suggesting broader implications of this drug class on AD pathology. Mouse models that exhibit both Aβ and tau pathology would provide crucial insights into the full therapeutic potential of SGLT2 inhibition in AD. Third, while we demonstrated cognitive improvements and elucidated several underlying molecular changes, the temporal sequence of these changes and their causal relationships require further clarification. Investigations of earlier timepoints could reveal the sequence of pathophysiological changes induced by SGLT2 inhibition. Additionally, studies examining the interaction between SGLT2 inhibitors and existing AD therapeutics could inform potential combination treatment strategies.

In conclusion, our findings demonstrate that SGLT2 inhibition exhibits significant therapeutic potential in AD through AMPK‐induced enhancement of microglial phagocytosis of Aβ, reduction of neuroinflammation, and improvement of synaptic function. The high potency and established safety profile of Enavogliflozin make it a promising candidate for clinical translation. Future studies addressing the outlined limitations and exploring potential combination therapies could help optimize its therapeutic application in AD treatment.

## Author Contributions

J.H. and I.M.‐J. conceived the project. J.H. and J.S. performed animal experiments. J.H. and J.S. wrote the manuscript. E.S.J., J.W.C., H.Y.J., and I.M.‐J. revised the manuscript. I.M.‐J. supervised the study.

## Conflicts of Interest

The authors declare no conflicts of interest.

## Supporting information


**Figure S1.** SGLT2 inhibition mitigates Aβ Pathology and neurodegeneration.
**Figure S2.** SGLT2 inhibition alleviates neuroinflammation.
**Figure S3.** Microglia show strong correlation with Aβ pathology.
**Figure S4.** Enavogliflozin is tolerable up to 30 μM in presence of Aβ.
**Figure S5.** SGLT2 inhibition reduces mitochondrial oxidative stress.
**Figure S6.** SGLT2 inhibition enhances microglial function in the 5XFAD mouse model.

## Data Availability

Data will be available from the corresponding author upon request.
