# Supplementary material for: SGLT2 Inhibition by Enavogliflozin Significantly Reduces Aβ Pathology and Restores Cognitive Function via Upregulation of Microglial AMPK Signaling in 5XFAD Mouse Model of Alzheimer's Disease
Source: Aging Cell. 2025 May 10;24(8):e70101. doi: 10.1111/acel.70101 (PMC12341776; doi:10.1111/acel.70101)
Supplement: Supplementary file 1 — Figure S1. SGLT2 inhibition mitigates Aβ Pathology and neurodegeneration. Figure S2. SGLT2 inhibition alleviates neuroinflammation. Figure S3. Microglia show strong correlation with Aβ pathology. Figure S4. Enavogliflozin is tolerable up to 30 μM in presence of Aβ. Figure S5. SGLT2 inhibition reduces mitochondrial oxidative stress. Figure S6. SGLT2 inhibition enhances microglial function in the 5XFAD mouse model. [file ACEL-24-e70101-s001.pdf]

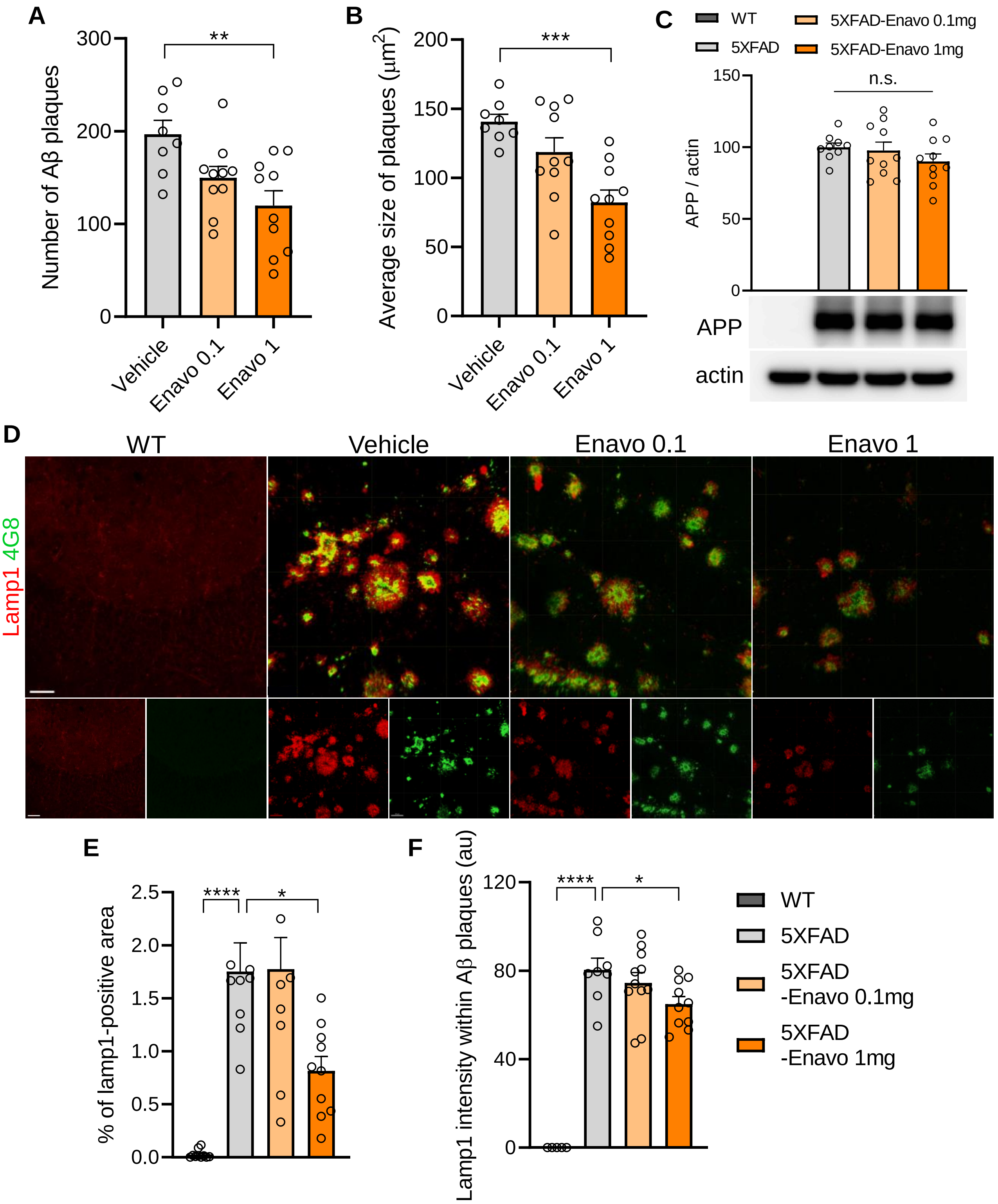

Supplementary Figure 1. SGLT2 inhibition mitigates Aβ Pathology and neurodegeneration.

(A) Quantification of total number of Aβ plaques in hippocampal sections of 7-month-old 5XFAD mice after 2 months of Vehicle or Enavogliflozin (0.1 μM, 1 μM) treatment. **$P < 0.01$. (B) Measurement of average plaque size (μm²). ***$P < 0.001$. (C) Representative Western blot images showing APP protein levels in WT and 5XFAD mice with or without Enavogliflozin treatment, and densitometric analysis of APP normalized to β-actin, n.s. = not significant. (D) Representative confocal microscopy images co-stained for Lamp1 (red) and 4G8 (green). Scale bar = 30 μm. (E) Quantification of Lamp1-positive area. *$P < 0.05$, ****$P < 0.0001$. (F) Quantification of Lamp1 intensity within Aβ plaques. *$P < 0.05$, ****$P < 0.0001$. All analyses were performed with 9-10 mice per group. Data are presented as mean ± SEM. Statistical analysis was performed using one-way ANOVA followed by Tukey's post-hoc test.

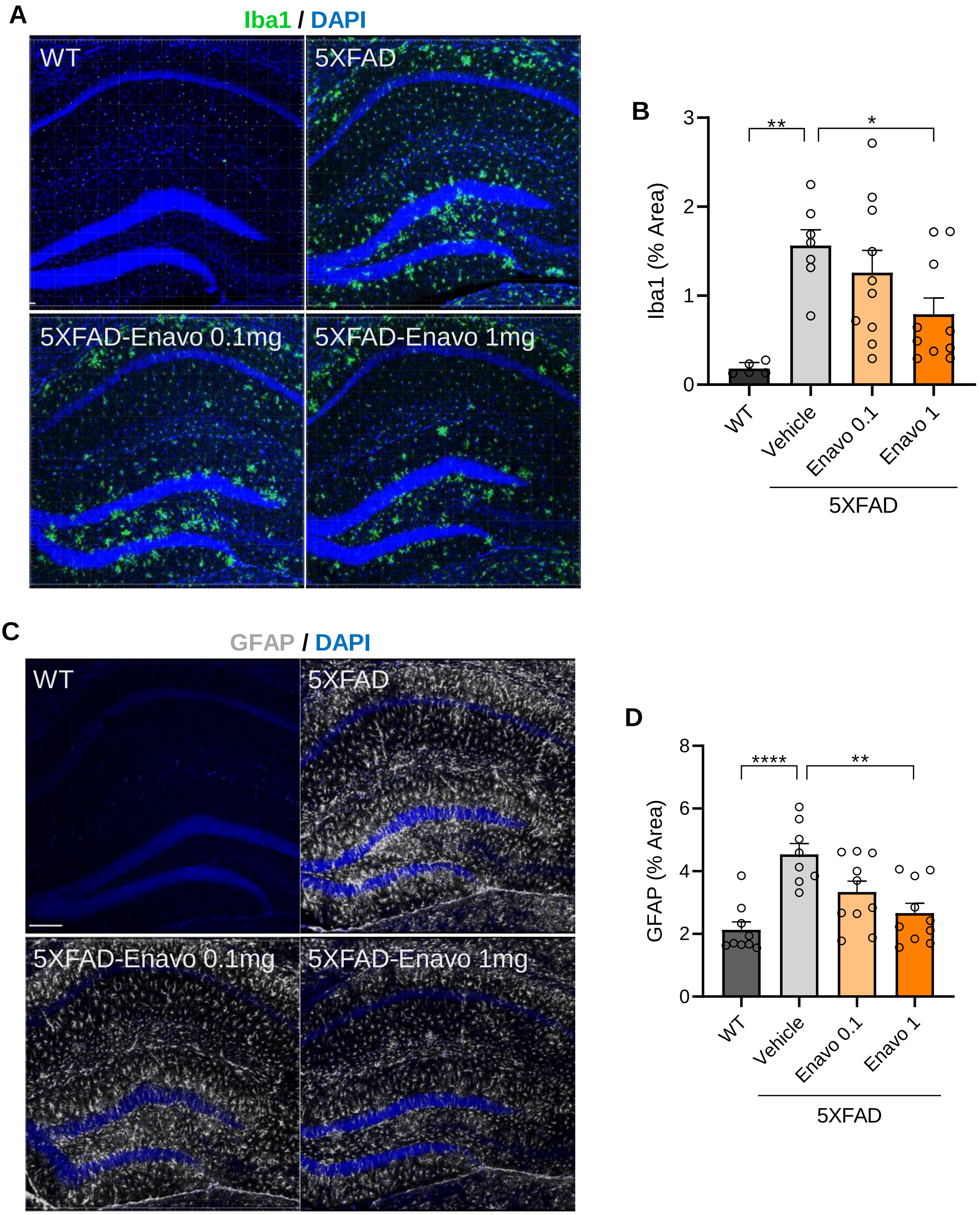

Supplementary Figure 2. SGLT2 inhibition alleviates neuroinflammation.

(A) Representative confocal microscopy images of hippocampal sections immunostained for Iba1 (green) and DAPI (blue) in WT and 5XFAD mice with or without Enavogliflozin treatment. Scale bar = 100 μm. (B) Quantification of Iba1-positive area, n = 8-10 per group. *P < 0.05, **P < 0.01. (C) Representative confocal microscopy images of hippocampal sections immunostained for GFAP (grey) and DAPI (blue) in WT and 5XFAD mice with or without Enavogliflozin treatment. Scale bar = 100 μm. (D) Quantification of GFAP-positive area, n = 8-10 per group. **P < 0.01, ****P < 0.0001. Data are presented as mean ± SEM. Statistical analysis was performed using one-way ANOVA followed by Tukey's post-hoc test.

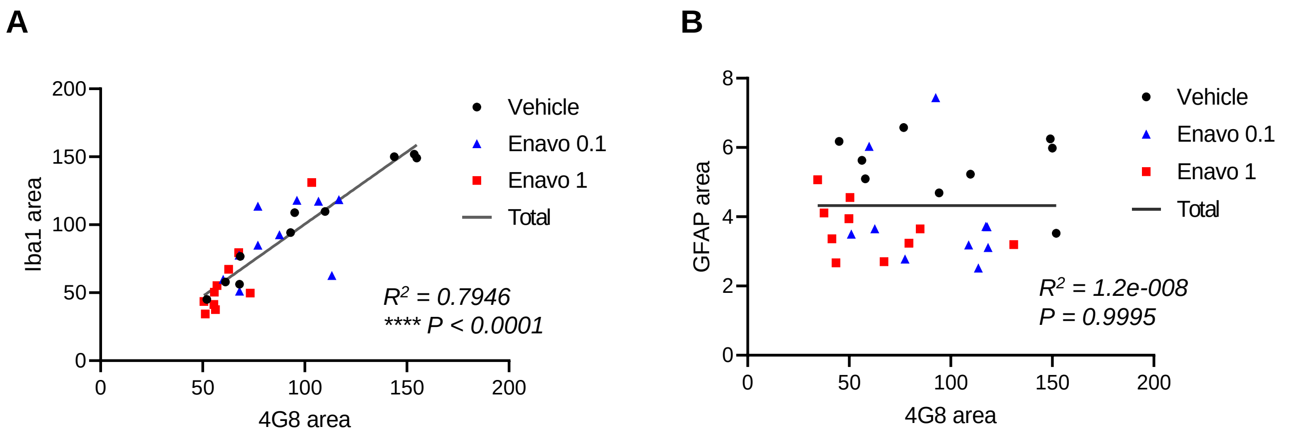

Supplementary Figure 3. Microglia show strong correlation with Aβ pathology.

(A) Scatter plot showing the correlation between 4G8-positive area and Iba1-positive area across all experimental groups. Each data point represents an individual mouse (black circles: Vehicle, blue triangles: Enavo 0.1, red squares: Enavo 1). Pearson correlation coefficient $R^2 = 0.7946$, ****$P < 0.0001$. (B) Scatter plot showing the correlation between 4G8-positive area and GFAP-positive area across all experimental groups. Each data point represents an individual mouse (black circles: Vehicle, blue triangles: Enavo 0.1, red squares: Enavo 1). Pearson correlation coefficient $R^2 = 1.2e\text{-}008$, $P = 0.9995$. Data were analyzed using Pearson correlation analysis.

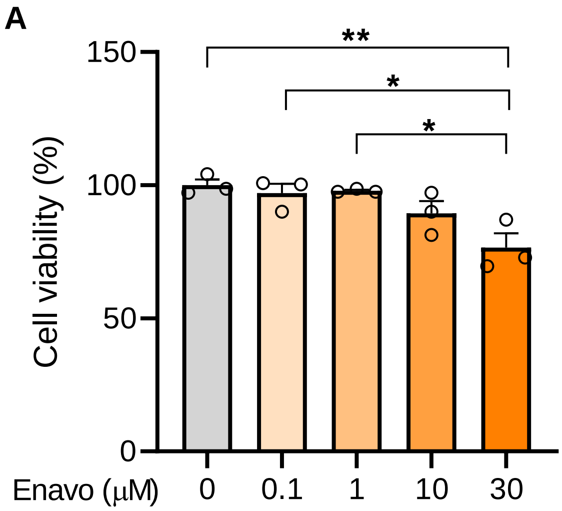

Supplementary Figure 4. Enavogliflozin is tolerable up to 30 μM in presence of Aβ.

(A) Cell viability of primary microglia exposed to increasing concentrations of Enavogliflozin (0, 0.1, 1, 10, and 30 μM) in the presence of Aβ for 24 hours, assessed using MTS assay, from 3 independent experiments. *P < 0.05, **P < 0.01. Data are presented as mean ± SEM. Statistical analysis was performed using one-way ANOVA followed by Tukey's post-hoc test.

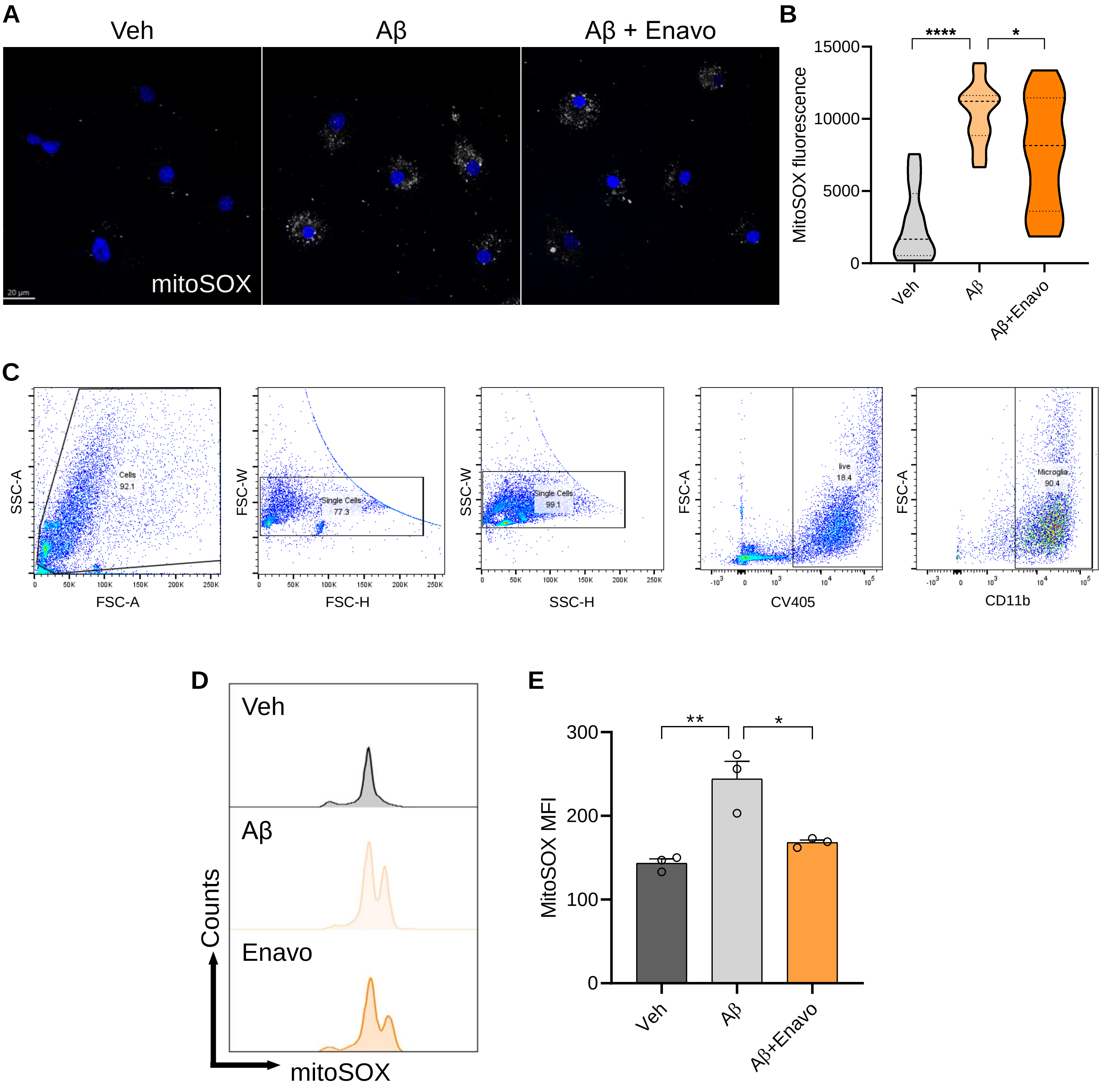

Supplementary Figure 5. SGLT2 inhibition reduces mitochondrial oxidative stress

(A) Representative confocal microscopy images of primary microglia treated with vehicle, Aβ (4 µM), or Aβ with Enavogliflozin (0.1 µM) for 24 hours and stained with mitoSOX (white) to detect mitochondrial superoxide production. Scale bar = 20 µm. (B) Quantification of mitoSOX fluorescence intensity from each cell of 4 independent experiments. *P < 0.05, ****P < 0.0001. (C) Representative flow cytometry gating strategy for analysis of primary microglia stained for mitoSOX. (D) Representative FACS profiles of primary microglia treated with vehicle, Aβ, or Aβ with Enavogliflozin populations for mitoSOX intensity. (E) Quantification of mitoSOX mean fluorescence intensity (MFI) measured by flow cytometry, from 3 independent experiments. *P < 0.05, **P < 0.01. Data are presented as mean ± SEM. Statistical analysis was performed using one-way ANOVA followed by Tukey's post-hoc test.

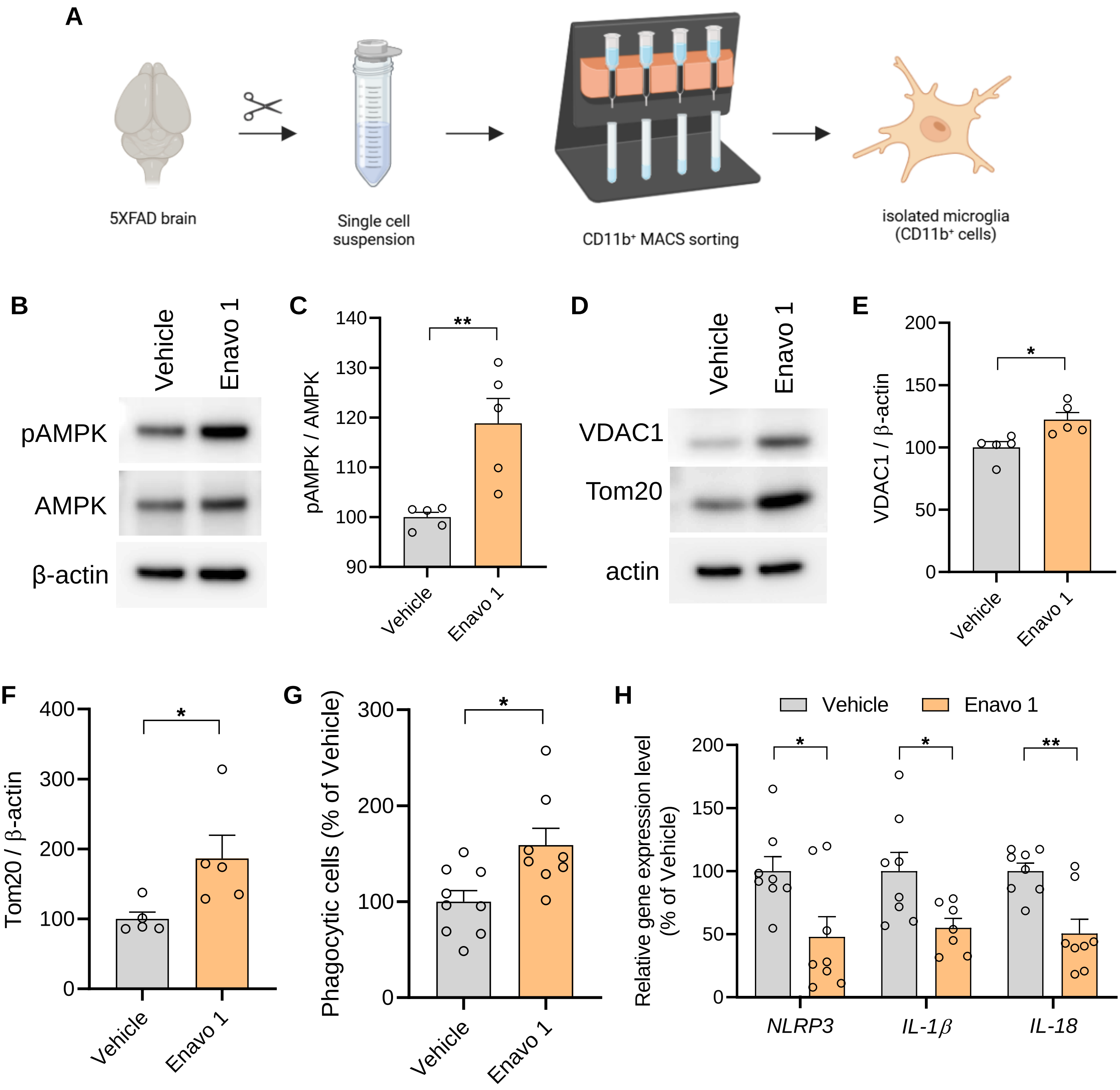

Supplementary Figure 6. SGLT2 inhibition enhances microglial function in the 5XFAD mouse model

(A) Schematic illustration of CD11b-positive microglia isolation from 5XFAD mouse brains. (B) Representative Western blot images showing phosphorylated AMPK (pAMPK), total AMPK, and β-actin in isolated microglia from vehicle or Enavogliflozin-treated mice. (C) Densitometric analysis of pAMPK to AMPK ratio, n = 5 per group. **P < 0.01. (D) Representative Western blot images showing VDAC1, Tom20, and actin protein levels in isolated microglia. (E-F) Densitometric analysis of VDAC1 (E) and Tom20 (F) normalized to β-actin, n = 5 per group. *P < 0.05. (G) Quantification of phagocytic cells measured by flow cytometry analysis of bead-positive microglial population, expressed as percentage of vehicle control, n = 8-9 per group. *P < 0.05. (H) Relative gene expression levels of NLRP3, IL-1β, and IL-18 in isolated microglia from vehicle or Enavogliflozin-treated mice measured by qRT-PCR, n = 8 per group. *P < 0.05, **P < 0.01. Data are presented as mean ± SEM. Statistical analysis was performed using unpaired Student's t-test.
